# Material Properties and Cellular Toxicity of 3D Scaffolds Based on Poly(ester-urethane) and Titania/Titanate Nanotubes

**DOI:** 10.3390/jfb17100514

**Published:** 2026-10-12

**Authors:** Dulce María González-García, Lucía Téllez-Jurado, Rodrigo Navarro Crespo, Alejandra Rubio Hernández-Sampelayo, María Rosa Aguilar, Rosa A. Ramírez, Ángel Marcos Fernández

**Affiliations:** 1 Depto. Ing. en Metalurgia y Materiales, Escuela Superior de Ingeniería Química e Industrias Extractivas (ESIQIE), Instituto Politécnico Nacional, (Unidad Profesional Adolfo López Mateos) UPALM-Zacatenco, Col. Lindavista, Mexico City 07738, Mexico; dgonzalezg@ipn.mx; 2 Institute of Polymer Science and Technology (ICTP-CSIC), Juan de la Cierva, 3, 28006 Madrid, Spain; rnavarro@ictp.csic.es (R.N.C.); alejandra@ictp.csic.es (A.R.H.-S.); mr.aguilar@ictp.csic.es (M.R.A.); raramirez@ictp.csic.es (R.A.R.); 3 Centro de Investigación Biomédica en Red de Bioingeniería, Biomateriales y Nanomedicina (CIBER-BBN), 28029 Madrid, Spain

**Keywords:** 3D scaffolds, freeze-drying, salt-leaching, polyurethanes, titania/titanate nanotubes, bone regeneration

## Abstract

The literature on techniques for creating 3D scaffolds in tissue engineering (TE) includes methods such as electrospinning, solvent casting, freeze-drying, salt leaching, and 3D printing, among others. These techniques, used individually or in combination, produce suitable scaffolds from different materials for the reconstruction of damaged tissues. The search for materials that fulfill the function of the tissues to be reconstructed makes the design, development, and evaluation of their physicochemical properties a relevant topic of study in tissue engineering. In this work, scaffolds with three-dimensional structures based on poly (ester-urethane) and a mixture of titania and titanate nanotube (TNT) composites (1, 5, and 10 wt.% of nanotubes) were obtained. The materials showed characteristic bands due to both polymeric and titania/titanate phases (observed by Raman and FT-IR spectroscopy). Scanning Electron Microscopy (SEM) analysis showed irregular porosity with pore sizes ranging from 8 to 700 µm. Also, mechanical testing showed tensile strength values of 3.3 to 5.31 MPa and Young’s modulus values of 18 to 28.8 MPa. The scaffolds were produced by a combination of freeze-drying followed by salt leaching techniques. In vitro biological assays using human osteoblast-like cells demonstrated that the materials were non-cytotoxic and supported cell viability, with excellent cell attachment and thermal stability for their intended application in tissue regeneration.

## 1. Introduction

The need to rebuild or replace damaged tissue in the human body has led to the development of tissue engineering (TE) and regenerative medicine (TERM), a discipline based on the interaction of three elements: stem or progenitor cells. These cytokines induce cell differentiation and/or proliferation, and scaffolds built from biopolymers organize or support the cells. One of the most demanding applications for biomaterial design is the repair of bone defects caused by genetic diseases or trauma when the bone is unable to regenerate on its own [1,2,3].

Bone is a specialized type of connective tissue that continually remodels in response to mechanical and hormonal stimuli. Its primary functions include providing structural support for soft tissues, storing ions such as calcium and phosphate, and protecting organs within the body. Additionally, bone serves as an attachment point for muscles, making it an essential tissue for locomotion. Furthermore, long and flat bones harbor bone marrow cells [4,5]. Due to these important functions, bone is considered essential for the proper functioning of the human body.

Bone is known as a composite material of both organic and inorganic components joined by weak interactions such as hydrogen bridges and van der Waals forces. The inorganic matrix constitutes approximately 45 to 60 wt%, while the organic component comprises 20 to 30 wt%, and the remaining 10 to 20 wt% is water. The organic matrix provides bone resistance to tensile and bending forces, while the inorganic matrix provides resistance to the shear and compression forces of bone tissue. Tensile strength for cancellous bone, known as spongy bone, has been reported to range from 5 to 10 MPa [6,7].

Therefore, extracellular matrices intended to serve as a suitable scaffold for bone regeneration must meet several important requirements, such as being biocompatible, non-cytotoxic, and biodegradable in the human body so that they can be replaced as bone grows naturally, in addition to possessing sufficient mechanical properties to facilitate this process. Also, they could be viable for cell growth [8].

Several studies have investigated composites for bone tissue repair, focusing on different material combinations [9,10]. Among these, polyurethane-based composites have gained attention due to their multiple combinations and tailoring properties [10,11], such as the combination of polyurethanes with hydroxyapatite [12], titania, or carbon nanotubes [13]. These composites have demonstrated excellent mechanical properties and a good distribution of the inorganic component within the organic matrix. Some studies compare the mechanical properties of polymer matrices with different chemical compositions and also explore the effect of adding nanoparticles at different concentrations, highlighting the importance of the mechanical properties of materials for tissue regeneration [14]. On the other hand, nanostructures such as titania nanotubes have been studied for bone tissue regeneration applications, demonstrating good osteogenic activity [15,16]. The morphology of titania nanotubes offers the advantage of a large specific surface area, which increases the contact area for enhanced cell integration. TNTs have demonstrated great potential to enhance the bioactivity and bone integration of the implants, promoting cell adhesion and proliferation. Their cylindrical directional structure provides physical guides that mimic the natural extracellular matrix, modulating cell behavior and differentiation [17]. Also, TNTs provide antibacterial properties and protection against infections, depending on their surface topography and biofunctionalization [18,19]. Furthermore, this morphology allows for efficient drug loading, storage, and controlled release [20]. This capability is substantially more efficient than other allotropic forms of TiO_2_ [18]. In addition, the chemical modification of TNTs’ surfaces allows the incorporation of advanced functionalities, such as NIR-light-activated photocatalysis for controlled drug release, further expanding their therapeutic potential. These features clearly highlight the advantages of TNTs over other conventional forms of TiO_2_ [21].

However, not only does the selection of materials and their chemical nature influence tissue regeneration, but it is also essential that the geometry and morphology of the scaffolds mimic the host tissue. Therefore, many scaffold manufacturing techniques have been explored. The electrospinning method has been used to obtain highly flexible composites for the regeneration of soft tissues, such as muscles and tendons, among others. In contrast, methods like solvent-casting, freeze-drying [22], and 3D printing [23,24] are the main options for manufacturing composites for hard tissue regeneration, such as bone [22]. The combination of these techniques can lead to the development of materials with a high porous distribution with regular shapes. There are numerous studies on 3D scaffolds based on composite materials for bone regeneration [25,26,27], some of which are based on polyurethanes with titania nanoparticles, demonstrating that these titania nanoparticles greatly influence chemical and biological behavior [28,29,30].

According to the literature review, studies on obtaining 3D polyurethane-based scaffolds with titania/titanate nanotubes using a combination of freeze-drying followed by salt leaching techniques for their possible application in tissue engineering are limited. Therefore, the present work analyzes the preparation and cytotoxicity of composite materials and their properties and proposes obtaining 3D scaffolds based on poly(ester-urethane) and titania/titanate nanotubes by freeze-drying after salt leaching techniques.

## 2. Materials and Methods

### 2.1. Materials

Polycaprolactone (PCL) diol with a molecular weight of 1000 g·mol^−1^ was purchased from Perstorp (Malmö, Sweden). 1,4-Butanediol (BD) and 1,6-diisocyanatohexane (HDI) were used in the synthesis of poly(ester-urethane) and purchased from Sigma-Aldrich Química S.L (Madrid, Spain). N, N-Dimethylacetamide (DMAc) was used as a solvent, while tin (II) 2-ethylhexanoate (Sn (Oct)_2_) was used as a catalyst, both purchased from Sigma-Aldrich Química S.L. (Madrid, Spain). The PCL diol was vacuum-dried at 80 °C, and both HDI and DMAc were purified by distillation prior to the polyurethane synthesis reaction. Additionally, titania nanopowder (TiO_2_), 1,1,1,3,3,3-hexafluoroisopropanol (HFIP), and dimethylformamide (DMF) were purchased from Aldrich. Potassium hydroxide (KOH) and hydrochloric acid (HCl) were purchased from Fermont (Monterrey, Mexico).

### 2.2. Methods

#### 2.2.1. Titania Nanotubes Synthesis (TNTs)

Titania nanotubes were synthesized using the magnetic stirring method previously reported by Alam Khan et al. [31]. An adequate amount of titania nanopowder (Aldrich, Naucalpan de Juárez, México), of 21 nm primary particle size) was combined with an 8 M potassium hydroxide (KOH) solution and mixed in a reaction flask. The mixture was heated at 120 °C for 12 h, using a continuous Argon gas flow to create an inert atmosphere. After the reaction, the product was filtered and washed with a 0.2 M hydrochloric acid (HCl) solution and then with deionized water until a neutral pH was achieved. Finally, the product was dried at 100 °C for 24 h.

#### 2.2.2. Poly(ester-urethane) Synthesis (SPU)

The poly(ester-urethane) (SPU) was synthesized using a two-step method. In the first step, polycaprolactone diol was charged into a reaction flask with 1,6-diisocyanatohexane (HDI) and Sn (Oct)_2_ (0.1 wt% with respect to the total polymer weight) and allowed to react at 80 °C for 4 h. In the second step, 1,4-butanediol (BD) was added, and the reaction was carried out at 80 °C for 4 h. During the reaction, 50 mL of N,N-dimethylacetamide (DMAc) was added as a solvent.

The reactant ratio of poly(ester-urethane) components was determined to obtain a hard segment content of 30 wt%, as previously reported [1].

#### 2.2.3. Composites Preparation

An appropriate amount of segmented poly(ester-urethane) (SPU) was dissolved in 1,1,1,3,3,3- hexaflouroisopropanol (HFIP). Next, titania nanotubes were dispersed at concentrations of 1, 5, and 10 wt% relative to the weight of SPU. The resulting solution was cast into Teflon containers and dried at room temperature for 24 h. Afterward, the films were vacuum-dried for 24 h to remove any solvent residue. Finally, the films were used for tensile strength testing (ISO 37 standard) [32].

#### 2.2.4. Preparation of 3D Scaffolds. Freeze-Drying Followed by Salt Leaching Techniques

The SPU was previously dissolved in dimethylformamide (DMF) using a ratio of 1 g/4 mL and heated at 80 °C. Once dissolved, 0.10 mL of distilled water was added, causing the SPU to precipitate into small spheres, which were then magnetically stirred until they were completely dissolved again. Water was added to lower the solution’s freezing point and create pores.

To prepare the SPU/TNTs composite scaffolds, the corresponding weight of TNTs (1, 5 and 10 wt %) was dispersed in DMF for 30 min and then added to the previously prepared SPU solution. Finally, 0.8 g of pre-sieved sodium chloride (NaCl) (particles not higher than 200 microns) was added and stirred until the product was homogenized.

The mixture was frozen in liquid nitrogen for several minutes, then transferred to a refrigerator at −30 °C for 1 h, and subsequently it was frozen again in liquid nitrogen. The above procedure was repeated 3 times. Finally, the solvent and water were freeze-dried at 0.045 mbar with a condenser temperature of −70 °C for 24 h. The resulting 3D scaffolds were washed with ethanol for 1 h, then with water for 3 days to remove the NaCl.

### 2.3. Characterization Techniques

The average molecular weight of SPU was determined by size exclusion chromatography (SEC) in a Perkin-Elmer series 200 gel chromatograph (Perkin-Elmer, Shelton, CT, USA). Water columns of 10^4^, 10^3^, and 100 Å, at 35 °C, were used, with a flow rate of 1 mL min^−1^ and DMF (HPLC-grade) as the solvent. Also, the polystyrene standard was used for calibration.

Structural analysis by Raman spectroscopy was performed using a Renishaw InVia Reflex instrument (Renishaw plc, Gloucestershire, UK) equipped with a diode laser of wavelength 785 nm.

Fourier transform infrared spectroscopy (FT-IR) analysis was performed in a Perkin-Elmer Spectrum Two spectrometer (Perkin-Elmer, Shelton, CT, USA ) coupled with an attenuated total reflection accessory (Perkin-Elmer ATR) at room temperature. FT-IR spectra were recorded in the mid-range between 4000 and 400 cm^−1^ with an average of 16 scans and a spectral resolution of 8 cm^−1^.

The crystal structure was determined by X-ray diffraction (XRD) using a Bruker D8 Advance diffractometer (Bruker, Billerica, MA, USA) with Cu kα radiation, in the 2θ range of 5° to 70° at a rate of 2°/min and a step size of 0.02°. Gaussian curves and the Origin program were used in order to do the deconvolution analysis for phase assignment.

Differential Scanning Calorimetry (DSC) was performed using a Mettler Toledo DSC 822e calorimeter (Mettler Toledo, Schwerzenbach, Switzerland) equipped with a liquid nitrogen accessory at a heating rate of 10 °C/min over a temperature range of −80 to 200 °C under a nitrogen atmosphere of 30 mL·min^−1^. Two thermal scans were recorded, and the glass transitions were recorded from the second scan, maintaining a rate of 10 °C/min during the cooling stage from 200 to −80 °C between the first and second heating runs. Thermogravimetric analysis (TGA) was carried out with a Mettler-Toledo TGA/SDTA 851 instrument (Mettler-Toledo, Schwarzenbach, Switzerland) from room temperature to 800 °C under a nitrogen atmosphere at a heating rate of 10 °C/min.

Scanning electron microscopy (SEM) and transmission electron microscopy (TEM) images of the TNTs were obtained using a JEOL 3600 for SEM and a JEOL JEM-2100 (JEOL Ltd., Tokyo, Japan) for TEM analyses. To report the average diameter sizes of TNTs, at least five SEM and TEM micrographs were evaluated using the FIJI program, with a standard deviation of ±5%. Furthermore, SEM images of the PEU/TNTs composites (3D scaffolds) were obtained using a Hitachi SU 800 microscope (Hitachi, Tokyo, Japan); the samples were previously well dried under vacuum and coated with gold by the sputtering method.

The obtained porosity of the scaffolds was theoretically calculated as in the literature [33]. Based on the SPU-salt ratio used to obtain scaffolds, with a theoretical density of 1.20 g/cm^3^ for the SPU and 2.165 g/cm^3^ for NaCl, the polymer mass was replaced with the mass of the washed scaffolds. The porosity was determined using gravimetric analysis of the average mass loss of five samples after washing. Also, to report the pore size distribution, SEM micrographs were analyzed using the FIJI program; about 500 pores were analyzed to report the data.

Tensile tests were conducted on an MTS Q test ^TM^ 1/L dynamometer, equipped with a 100 N load cell. The samples were die-cut into a dumbbell shape Type 4, according to the ISO 37 standard, using previously obtained PEU/TNT composite films. At least 6 specimens per composition were tested with a crosshead speed of 5 mm min^−1^. The strain was measured based on the crosshead separation from an initial length of 12 mm. All the mechanical and deviation values reported in this work, including Young’s modulus, were calculated by the dynamometer software (Testworks^®^4, version 4.06A).

### 2.4. Biological Studies

#### 2.4.1. Indirect Assay with Osteoblastic Type Cells

This assay was performed to study whether the scaffolds obtained in this work released toxic substances into the medium that could alter cell viability; the test was performed following the procedure reported in ISO 10993-5:2025 [34] and ISO 10993-12 [35]. Viability was measured quantitatively using the AlamarBlue^®^ reagent (Invitrogen, Waltham, MA, USA). 40 mg of each scaffold was placed in tubes containing 4 mL of osteoblast culture medium and incubated at 37 °C with shaking.

At least 4 replicates were tested and their extracts (1 mL) were collected at 1, 2, 7, 14, and 21 days and frozen at −20 °C. The collected volume was replaced with the same volume of medium at each time point. After 21 days, the cells were seeded in 96-well plates at a concentration of 90,000 cells/mL (100 μL/well) and expanded for 24 h at 37 °C and 5% CO_2_. The following day, the medium was removed, and then 100 μL of the previously prepared extracts were added to each well, and the plates were incubated for 24 h.

After this time, the extracts were removed from the plates, and 100 μL of 10% (*v*/*v*) AlamarBlue^®^ reagent in phenol red-free medium was added to each well. The plates were incubated for 3 h, and fluorescence was analyzed at an excitation wavelength of 560 nm and an emission wavelength of 590 nm using the Biotek Synergy HT plate reader (Biotek Instruments, Winooski, VT, USA). Tissue culture plastic (TCP) was used as a negative control. Cell cultures on composites were compared with those cultured on SPU in order to determine the effect of the TNTs. Statistical analysis was performed using a two-way ANOVA, followed by Tukey’s multiple comparisons post hoc test. Differences were considered statistically significant at *p* < 0.05.

#### 2.4.2. Direct Assay with Osteoblastic Type Cells

The direct cell viability assay was performed to determine osteoblasts’ behavior in contact with the scaffolds, determining their adhesion and proliferation over time. The in vitro biocompatibility of the scaffolds and cell proliferation were assessed using the Human Osteoblast cell line (HOB 406-05f, ECACC Sigma-Aldrich) at 1, 2, 7, and 14 days, changing the medium every 3 days with fresh medium. The scaffolds were previously sterilized in absolute ethanol for 1 h, followed by three washes with sterile PBS (Phosphate Buffered Saline, pH 7.4, Sigma) and culture medium for 30 min. The medium used was the INNOPROT Osteoblast Medium Kit, ref. P60119, supplemented only with the kit’s add-ins.

The cells were seeded at a density of 5 × 10^5^ cells/mL in 100 µL of micro-seeding medium, resulting in 50,000 cells per scaffold. The scaffolds were incubated at 5% CO_2_ and 95% humidity for 14 days, with the medium being renewed every 3 days. The quantitative analysis was performed using AlamarBlue^®^ reagent (Invitrogen, Waltham, MA, USA). It was prepared at a 10% concentration with phenol red-free medium. 500 µL were added to each scaffold, incubated for 3 h in a CO_2_ incubator, and then transferred to a 96-well plate. Fluorescence lectures were taken at an excitation wavelength of 560 nm and an emission wavelength of 590 nm. The analysis was performed using a Biotek Synergy HT instrument ((BioTek Instruments, Winooski, VT, USA). For each experimental condition, at least four biological replicates (*n* ≥ 4) were evaluated, and eight technical replicates were analyzed for each biological replicate. Statistical analysis was performed using a two-way ANOVA, considering TNT concentration and culture time as independent factors, followed by Tukey’s multiple comparisons post hoc test. Differences were considered statistically significant at *p* < 0.05 (*), *p* < 0.01 (** or ++) and *p* < 0.001 (*** or +++).

SEM micrographs of cells attached to the scaffolds were obtained following the procedure described below. The samples were washed in PBS, and only the adhered cells were fixed with 3.7% *w*/*v* paraformaldehyde for 2 h. Then, they were washed 3 times with distilled water and dried at 37 °C until completely dry. Finally, samples were coated with gold/palladium by the sputtering method, and cell morphology micrographs were acquired using a Phillips XL30 microscope (Amsterdam, Netherlands) at 25 kV.

## 3. Results

### 3.1. TNTs Characterization

**Figure 1:**
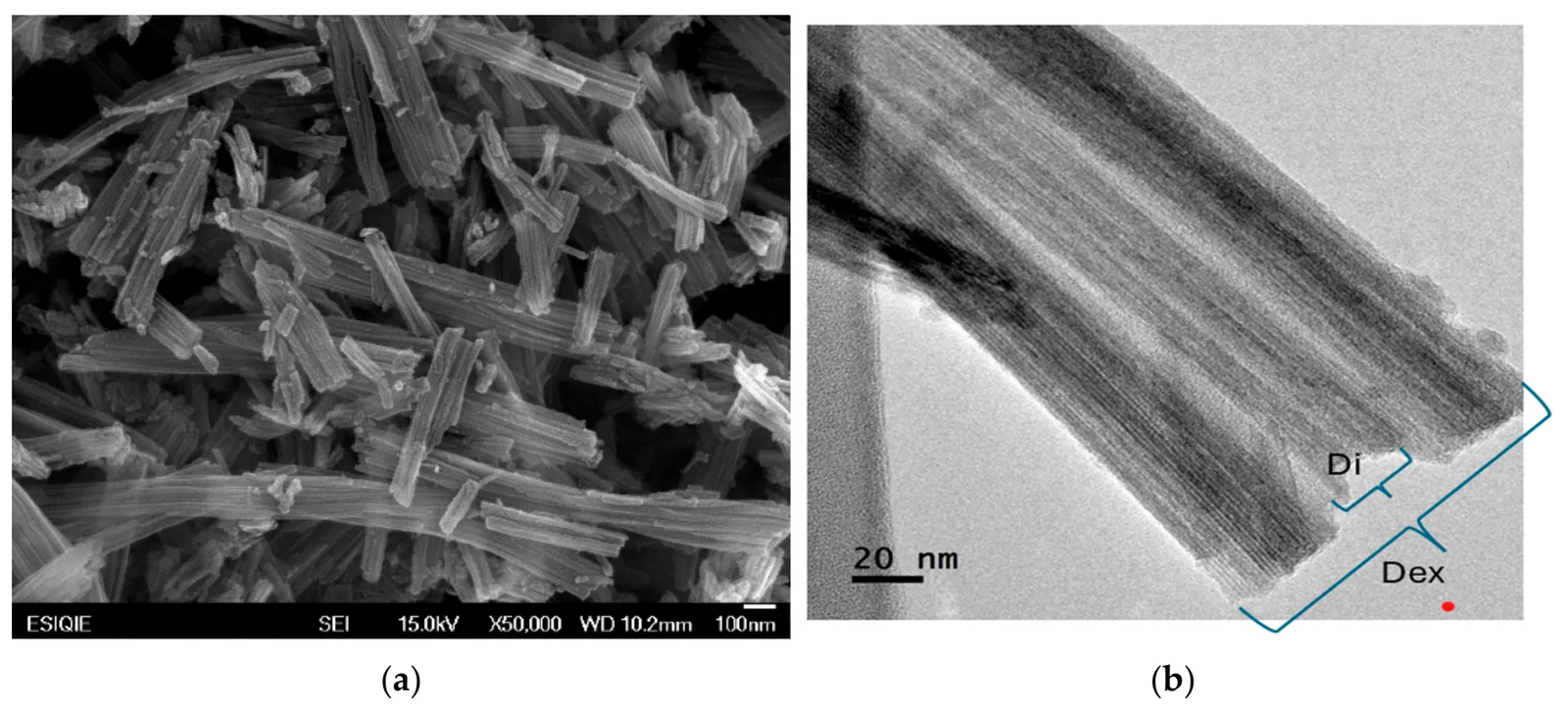

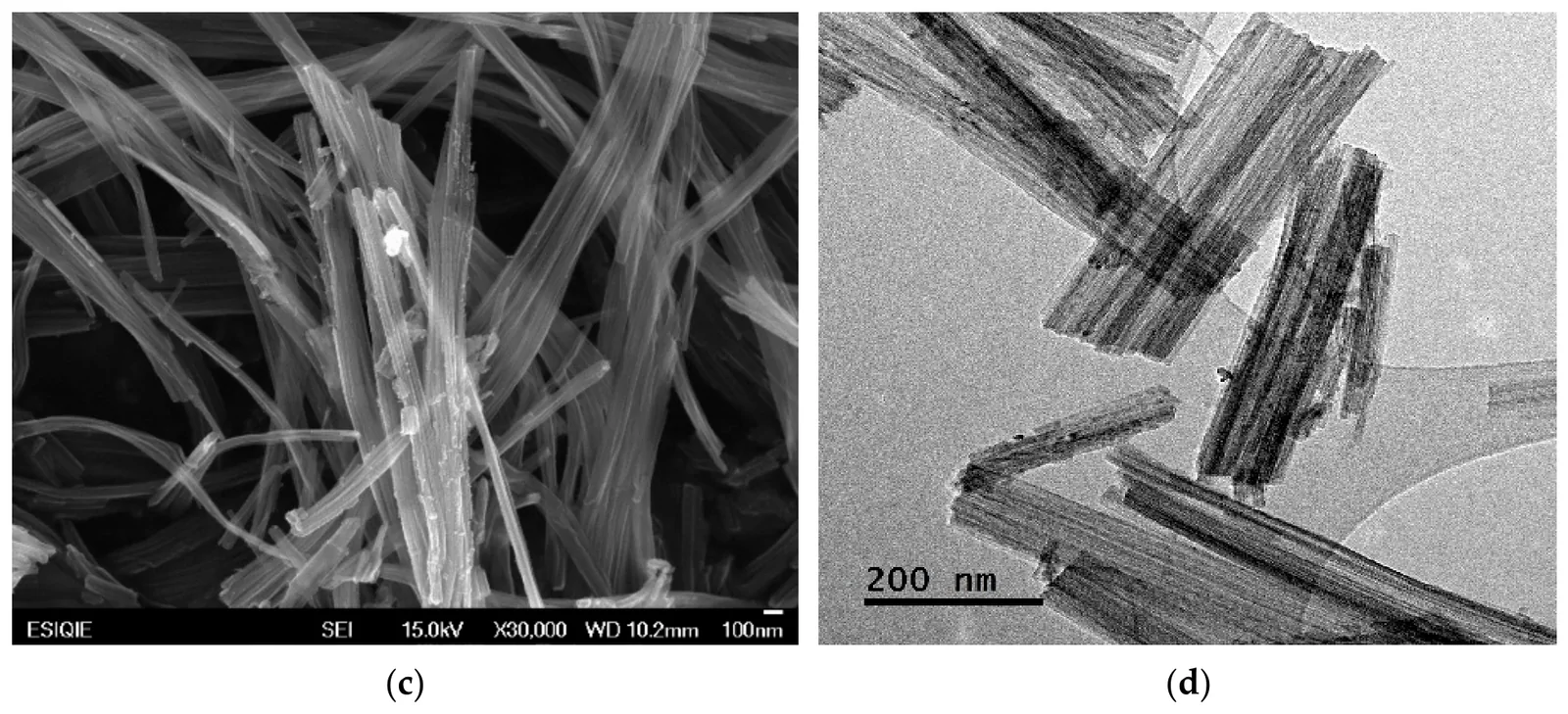
(**a**,**c**) SEM images and (**b**,**d**) TEM images of titania/titanate nanotubes (TNTs) obtained by the magnetic stirring method.

The SEM and TEM images of the titania/titanate nanotubes obtained by magnetic stirring are shown in Figure 1.

The images in Figure 1 show the tubular and elongated morphology of titania/titanate nanotubes (TNTs), which are randomly oriented and agglomerated with various nanotubes.

The average internal diameter of the TNTs ranged from 26 to 38 nm, and the average external diameter ranged from 80 to 95 nm. These internal sizes correspond to the nanotube sizes obtained by other authors [31,36]. These authors mentioned that internal diameters smaller than 100 nm belonged to tubular nanoparticles. The longitudinal sizes ranged from 250 to 2800 nm, and TEM images indicated that the nanotubes exhibited a multi-walled morphology, as described by other authors [31,36]. The internal and external diameters of the obtained titania nanotubes are shown in Figure 1c for reference.

**Figure 2:**
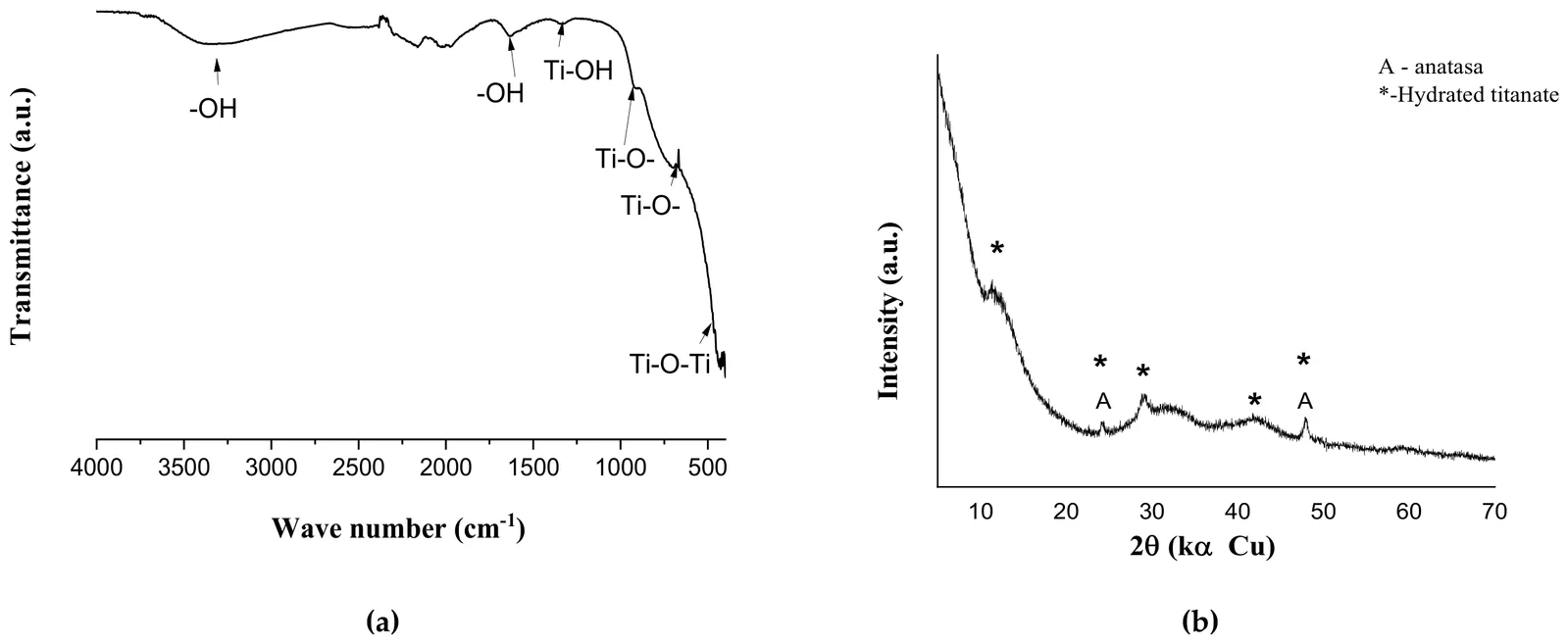
FT-IR spectrum (**a**) and XRD pattern (**b**) of the obtained TNTs.

Furthermore, it was mentioned that the presence of both rutile and anatase phases is a good combination because of their synergy, leading to better mechanical properties and chemical stability than titanium oxide with anatase or rutile phase alone [37,38]. Figure 2 presents the chemical and crystalline structure of the titania nanotubes, from the FT-IR (Figure 2a) and XRD analysis (Figure 2b), respectively.

FT-IR spectrum of the TNTs in Figure 2a, showed bands between 3500–3000 cm^−1^ and 1638 cm^−1^ related to the stretching and bending modes, respectively, of the (-OH) groups from water absorbed on the TNTs surface. The bands observed at 1336 cm^−1^, 918 cm^−1^ and 706 cm^−1^, and ~ 450 cm^−1^ are due to Ti-OH, Ti-O, and Ti-O-Ti bonds, respectively [39,40,41].

The XRD pattern of TNTs (Figure 2b) shows a mix of a hydrated titanate (H_2_Ti_4_O_9_·H_2_O) and a contribution of anatase according to JCPDS 36-655 and JCPDS 21-1272, respectively [42]. Additionally, a halo with low-intensity peaks was observed below 20° 2θ, and it corresponds to short-range structures or nanostructures [43]. Other authors [42] mentioned that the nanostructures obtained using an alkali medium similar to ours induce the conversion of those crystalline phases into amorphous titanate phases in the nanotubes, such as hydrogen titanate phases (H_2_Ti_4_O_9_*H_2_O, JCPDS no 36-655) and potassium titanates (K_2_.nTiO_2_(OH)). All the above results indicate that a mix of titania and titanate nanotubes (TNTs)was obtained.

### 3.2. Polyurethane and Composites Characterization

The molar ratio, molecular weight, and polydispersity of the synthesized poly(ester-urethane) (SPU) are shown in Table 1.

**Figure 3:**
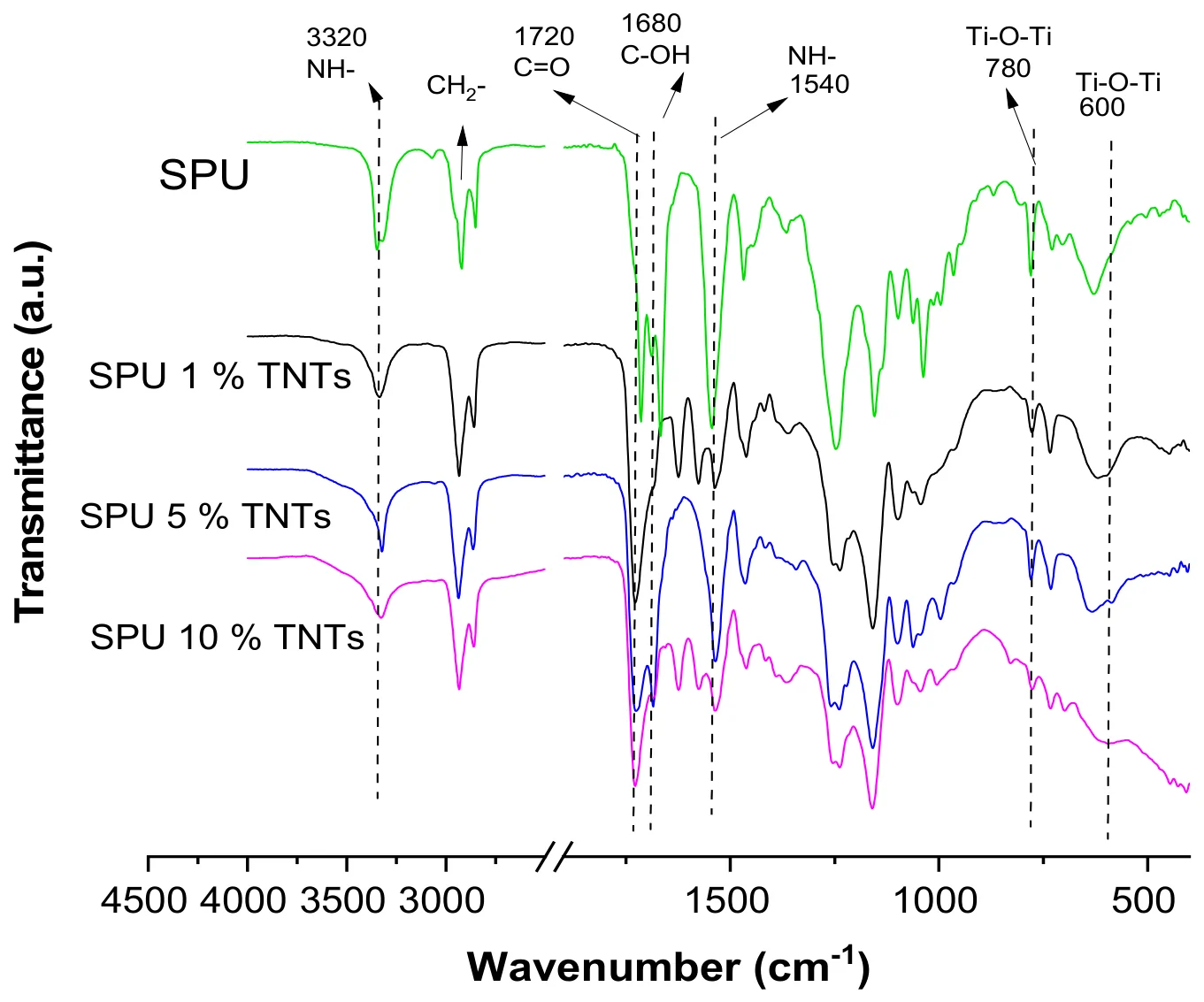
FTIR spectra of poly(ester-urethane) and composites with 1, 5, 10 wt% of TNTs.

The FT-IR spectrum of the SPU and composites is presented in Figure 3.

The FT-IR spectra in Figure 3 showed the main absorption bands due to the functional groups of the poly(ester-urethane). The band at 3320 cm^−1^ corresponds to the vibrations of the N-H stretching vibrations of the urethane groups. At 1720 cm^−1^, the band of free carbonyl groups (C=O) of esters from the soft segment (PCL) is observed, while the band at 1680 cm^−1^ is associated with hydrogen-bonded carbonyl groups [44,45,46]. For the composites of polyurethane and TNTs, it is observed that the band at 3320 cm^−1^ is widened and displaced; it could be related to the interaction by hydrogen bonds between the urethane group and the hydrated titanate (Ti-OH) [47,48]. Also, there are similar changes for the free carbonyl groups at 1720 cm^−1^, which corroborates the interaction between the nanostructures and the polymer matrix. The other significant changes in the spectra are the decrease in the intensity of -NH- groups at 1540 cm^−1^, related to amide groups of polyurethane, due to a possible crosslinking between the carbonyl of the amide groups and the titania/titanate nanotubes (TNTs). Finally, bands at 780 and 600 cm^−1^ of the carbon backbone of the polyurethane are overlapped by the Ti-O-Ti bands related to the TNTs [49,50].

**Figure 4:**
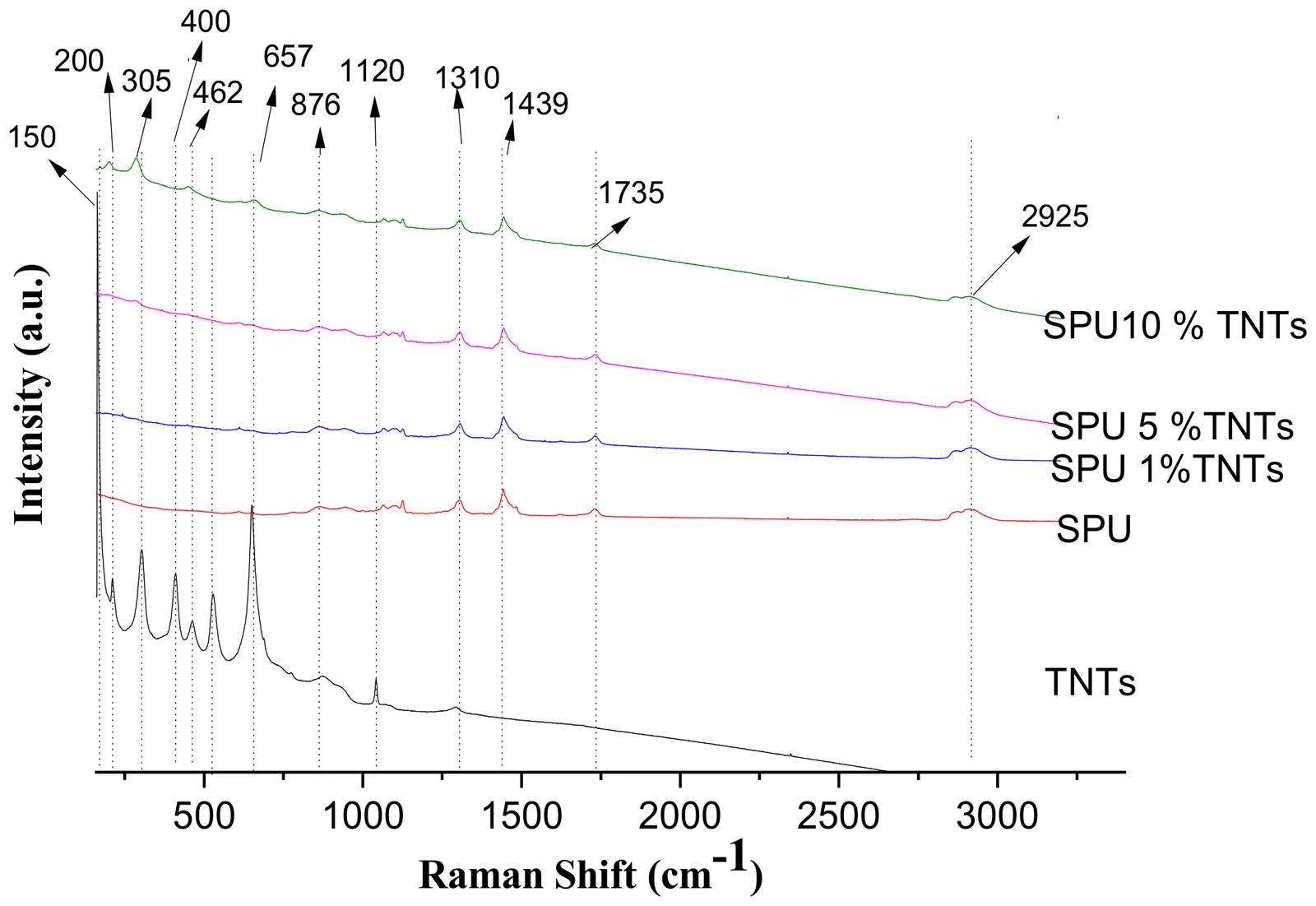
Raman spectra of poly(ester-urethane), TNTs, and composites.

Figure 4 presents the Raman spectra of TNTs, poly(ester-urethane), and their composites featuring 1, 5, and 10 wt% of TNTs.

The Raman spectra in Figure 4 show the spectral characteristics of each material mentioned earlier. Peaks associated with poly(ester-urethane) were found at 2925 cm^−1^ and 1310 cm^−1^, which were attributed to the ethylene (CH_2_-) groups in the polyurethane, and at 1735 cm^−1^ and 1439 cm^−1^, related to the -C=O and -NH bonds, respectively, while the bands ranging from 1120 cm^−1^ to 800 cm^−1^ were associated with the carbon skeleton of polyurethanes [51].

On the other hand, the Raman spectrum of TNTs reveals peaks corresponding to the crystalline phases of titania and rutile. The peaks at 190 cm^−1^ and 200 cm^−1^ corresponded to the tetrahedral structure due to [TiO_6_]. The peaks found at 305 cm^−1^, 410 cm^−1^, and 462 cm^−1^ were related to Ti-O-Ti bonds, and those at 525 cm^−1^ and 657 cm^−1^ corresponded to Ti-O bonds. Additionally, the peaks at 150 cm^−1^ and 410 cm^−1^ are assigned to the anatase phase, while the peaks at 462 and 655 cm^−1^ are associated with the rutile phase [52,53].

Finally, the Raman spectra of the composites displayed peaks related to both polyurethanes and TNTs. The peaks associated with TNTs became more pronounced as their content in the composites increased, and at 10 wt% of TNTs, the bands related to titania were more clearly observed [54,55].

**Figure 5:**
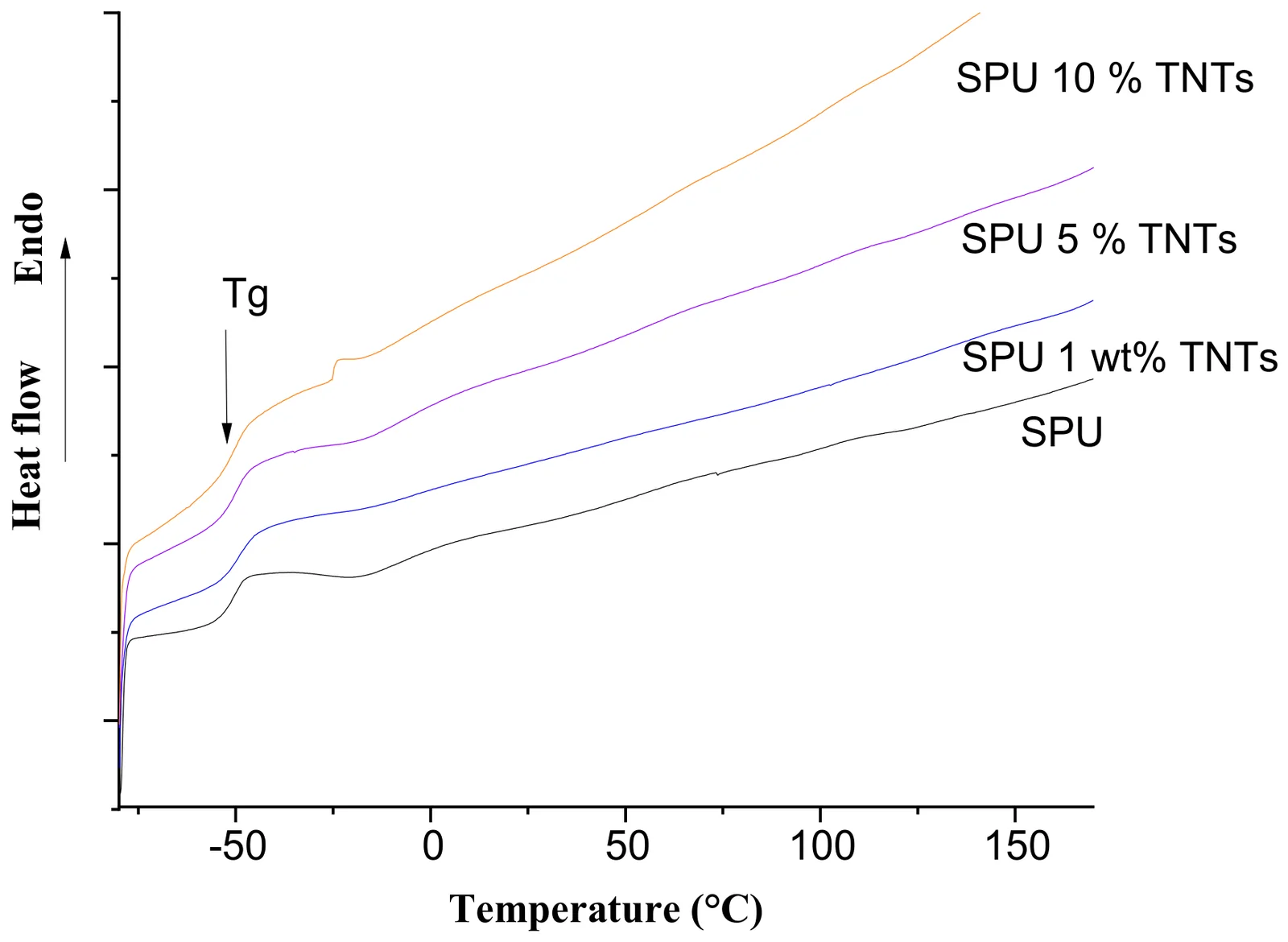
DSC of SPU and the composite materials with 1, 5, and 10 wt% of TNTs.

**Figure 6:**
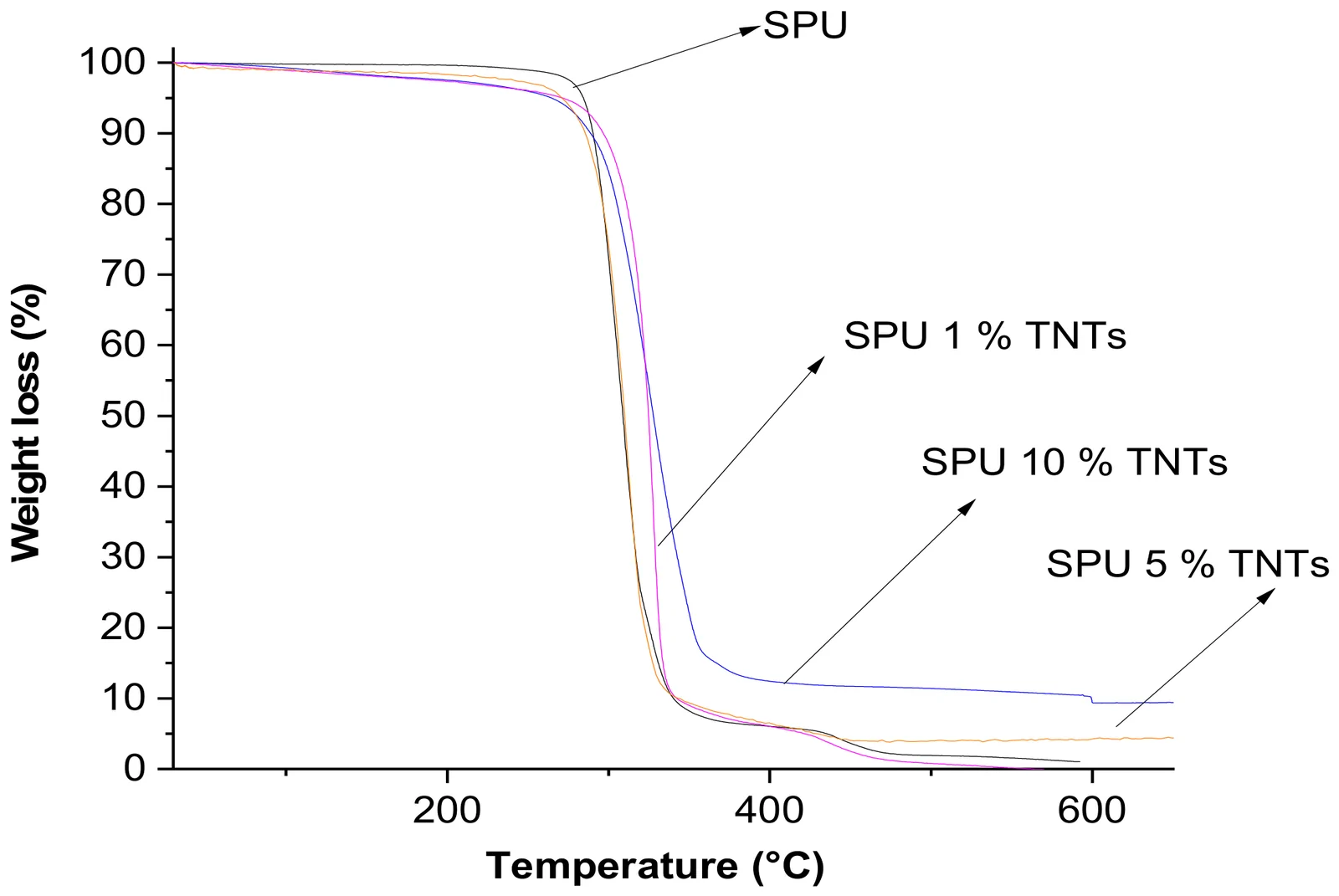
TGA of the poly(ester-urethane) and the composite materials.

The thermal behavior of the poly(ester-urethane) and the composites is illustrated in Figure 5, which shows the DSC curves, and in Figure 6, which presents the TGA analysis of the composite materials.

DSC analysis showed that the glass transition temperatures (Tg) of poly(ester-urethane) (SPU) and its composites were quite similar, ranging from −49 to −53 °C. The Tg of the SPU was recorded at −53 °C, while the composites containing 1%, 5%, and 10 wt% of TNTs exhibited Tg values of −49 °C, −50.1 °C, and −51.18 °C, respectively. As can be seen, the samples did not show crystallization or melting transitions, indicating their amorphous nature. Furthermore, the introduction of the TNTs into the polymer matrix did not affect Tg significantly, likely due to the low inorganic content. Jiawen Xiong et al. [56] analyzed polyurethane-based composite materials with carbon nanotubes and reported similar thermal behavior, with an increase in Tg upon incorporation of nanoparticles into the polymer matrix. They associated the shift in Tg temperatures with a possible crosslinking of the nanoparticles with the polyurethane matrix, thus hindering the mobility of the chains by these nanoparticles.

The TGA analysis shows that composite materials have similar thermal behavior regardless of the TNTs content. The small weight loss observed before 250 °C in the SPU 1 wt%, SPU 5 wt%, and SPU 10 wt% could be related to the presence of solvent trapped in the nanotubes. Also, a weight loss between 400 and 500 °C in all materials was related to the decomposition of carbon residues from the polymer [57]. Above 500 °C, a constant weight was maintained, related to the weight of the nanotubes added to the composite materials. The thermal behavior of the composites indicates that they are thermally stable materials. This result is highly relevant to ensure thermal sterilization and processing before their possible implantation.

**Figure 7:**
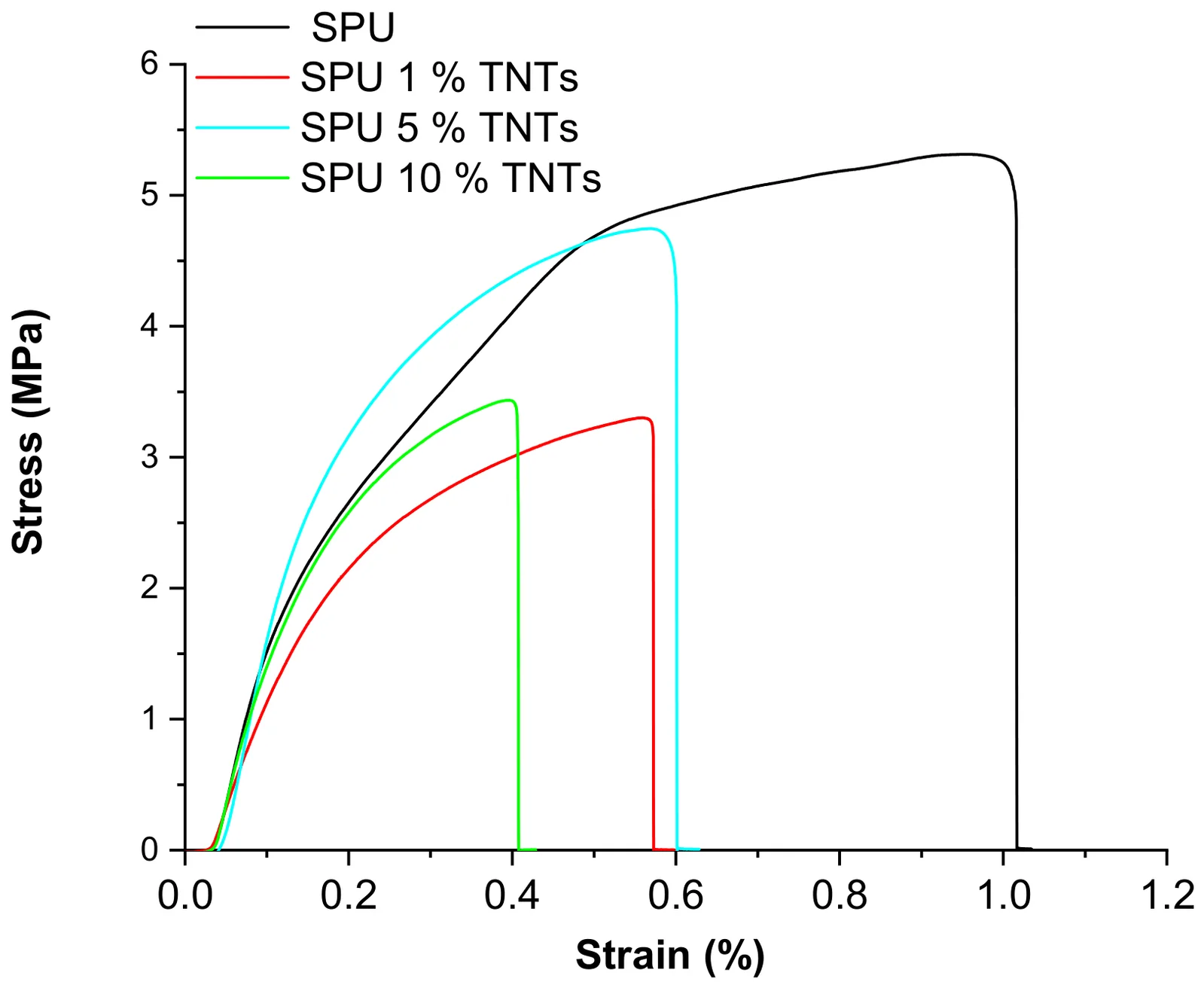
Stress-strain curves for poly(ester-urethane) (SPU) and the composites with 1, 5, and 10 wt% of TNTs.

To evaluate the mechanical behavior of the prepared materials, the tensile strength and Young’s Modulus of both the poly(ester-urethane) and the composites were assessed. Figure 7 presents representative stress-strain curves for the poly(ester-urethane) and the composites containing 1, 5, and 10 wt% of TNTs.

Figure 7 shows that the SPU had the highest strength, with a stress at break of 5.31± 0.8 MPa and a Young’s modulus of 27 ± 6.9 MPa. For the composites with 1%, 5%, and 10 wt% TNTs, stress and Young’s modulus values reached 3.3± 0.29 and 18 ± 1.37 MPa, 4.8 ± 0.3 and 28.8 ± 2.0 MPa, and 3.4 ± 0.3 and 26 ± 3.4 MPa, respectively. The incorporation of TNTs into the polymer matrix decreased the maximum stress achieved, which could be due to the randomly distributed TNTs acting as points of stress concentration, leading to premature rupture. No significant differences were found for Young’s modulus. Thus, the number of rigid particles in the composite seems not to be sufficient to affect the overall rigidity of the material. Also, a decrease in the percentage of deformation of the composite materials was observed due to the introduction of nanotubes into the polymer matrix. Other studies [58,59] on polyurethanes with similar ratios of carbon nanotubes reported similar behavior, with a decrease in the maximum strain values at higher content of nanotubes and no significant change in Young’s modulus. It must be noted that the intended application of those reports is different. The composites obtained in this work are suitable as base materials for scaffolds, since the stress values of cancellous trabecular bone are between 2 and 20 MPa [60,61].

### 3.3. Morphological and Biological Evaluation

**Figure 8:**
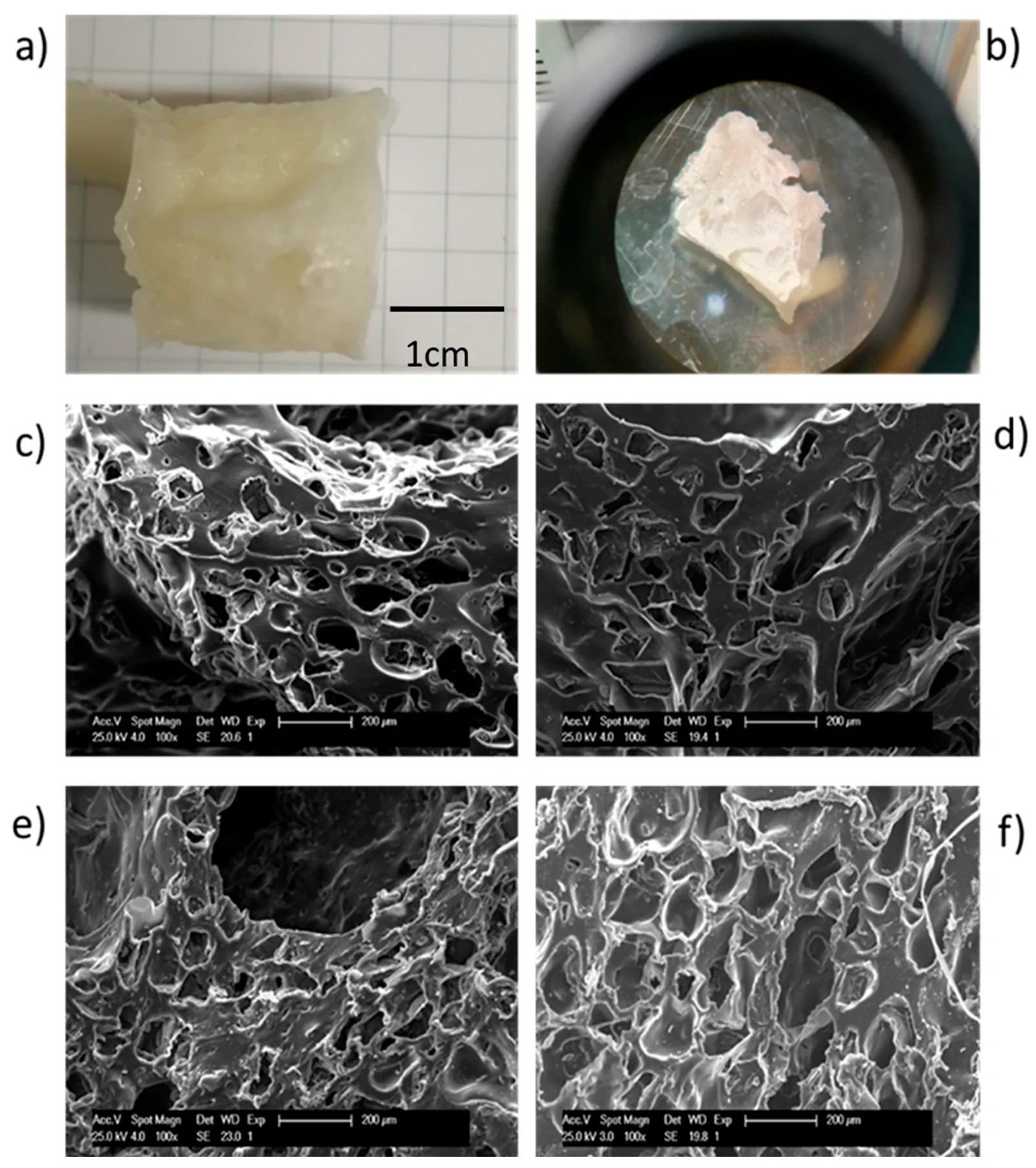
(**a**) Macrostructure of the 3D scaffolds, (**b**) Transversal cut of the 3D scaffolds, (**c**) SEM image of SPU scaffold, (**d**) SEM image of SPU 1 wt% TNTs scaffold, (**e**) SEM image of SPU 5 wt% TNTs scaffold, and (**f**) SEM image of SPU 10 wt% TNTs scaffold.

The geometry and morphology of scaffolds are important aspects for mimicking the host tissue. The obtained 3D scaffolds exhibited a pore structure because of the combination of two common techniques (lyophilization and salt leaching), as described in the experimental section. Figure 8 shows the macro- and micro-morphology of the scaffolds obtained.

**Figure 9:**
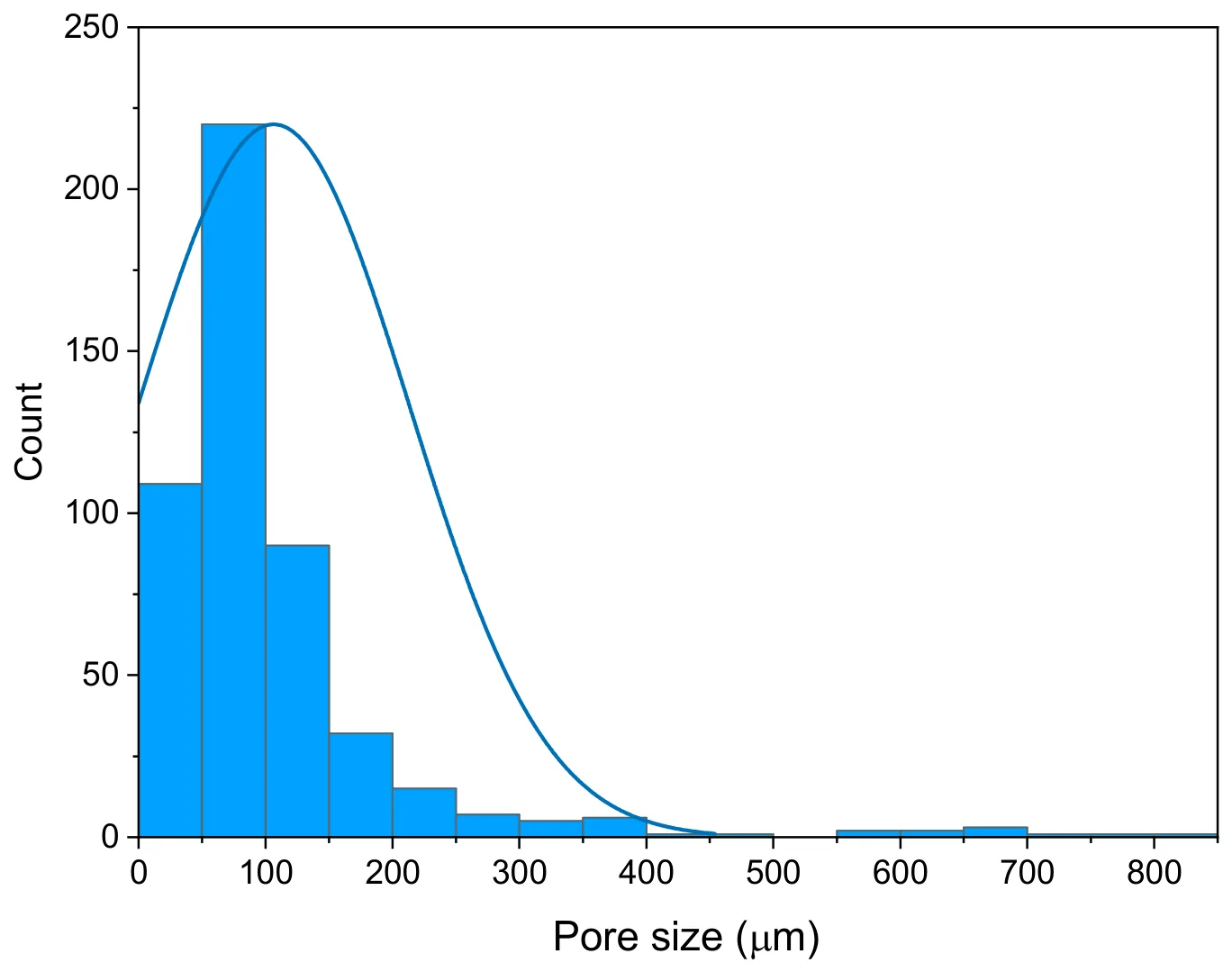
Pore size range and distribution in scaffolds.

The macrostructure showed a well-formed, highly porous sponge-like structure. SEM images of the 3D scaffolds showed irregular pores, with sizes ranging from <20 to 700 µm. The obtained porosity was calculated to be about 58% ± 3%. Also, the pore size distribution was calculated as described in the methods section. Figure 9 shows the pore size distribution.

Figure 9 shows that the main pore size distribution ranges from 50 to 100 µm and from <20 to 50 µm, similar to those reported in the literature [62,63]. The titania/titanate nanotubes are not visible in the SEM images in Figure 8. This is attributed to their small quantity within the composites and their embedding in the polymer matrix. However, the presence of these nanostructures in the scaffold is confirmed by the TGA analysis.

**Figure 10:**
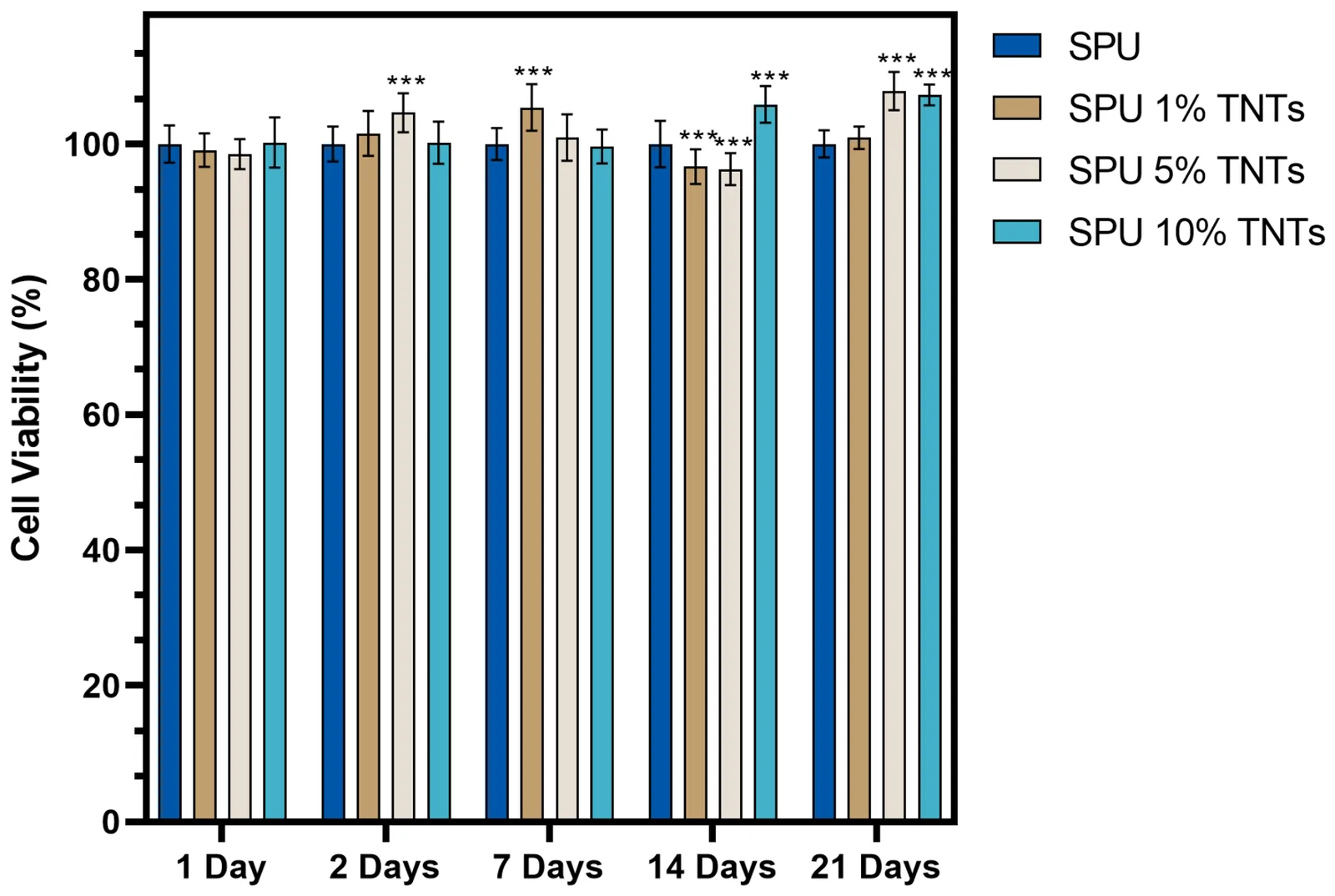
Indirect Alamar Blue assay: Metabolic activity (%) determined following exposure to lixiviates from polyurethane sponges (SPU) and titania/titanate nanotube-containing sponges (TNTs; 1%, 5%, and 10%). Data are presented as mean ± SD. The asterisk (*) indicates statistically significant differences compared with the SPU control at the corresponding time point (*** *p* < 0.001).

**Figure 11:**
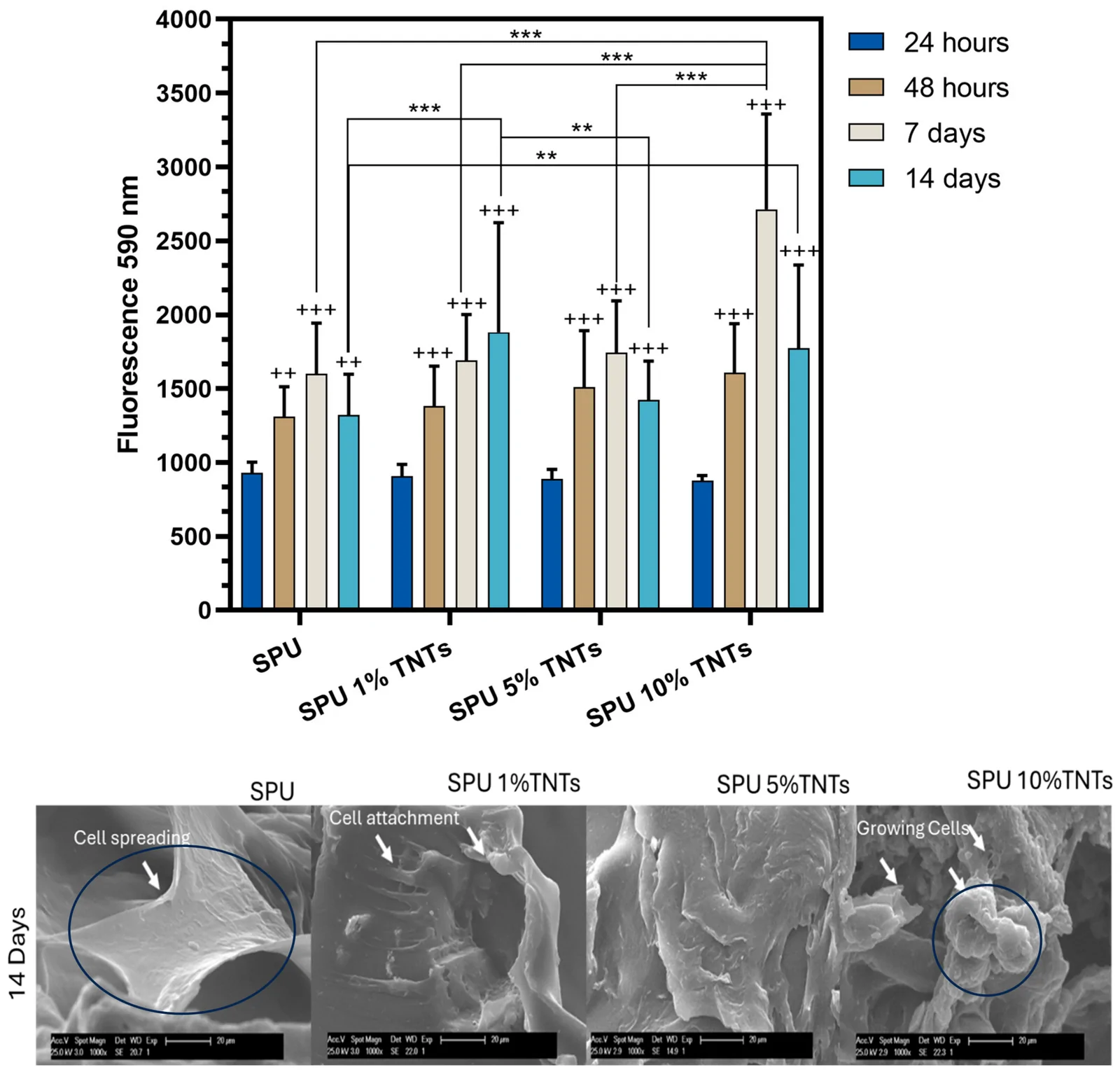
**Top:** Direct Alamar blue assay performed on polyurethane sponges (SPU) and polyurethane sponges containing titania/titanate nanotubes (TNTs; 1%, 5% and 10%) after 24 h, 48 h, 7 days and 14 days of culture. Data are presented as mean ± SD. Statistical analysis was performed using two-way ANOVA to evaluate the effects of scaffold composition (*) and culture time (+). Significance levels are indicated as ** *p* < 0.01, *** *p* < 0.001 for composition-dependent effects and ++ *p* < 0.01, +++ *p* < 0.001 for time-dependent effects on metabolic activity. **Bottom**: The images show cell proliferation and attachment on the obtained scaffolds.

Finally, the in vitro biological assays were performed to determine the non-cytotoxicity of the scaffolds obtained in this work and the proliferation of human osteoblast-like cells (HOB). Figure 10 shows the indirect Alamar Blue assay. Figure 11 shows the direct Alamar Blue assay and the morphology of cells within the scaffolds.

The indirect Alamar Blue assay showed that cell viability remained close to or above 100% for all groups throughout the study, demonstrating the absence of cytotoxic effects associated with either the polyurethane matrix or the incorporation of TNTs. All measured values remained well above the 70% viability threshold established by ISO 10993-5 [34] for non-cytotoxic materials. These findings indicate that no toxic compounds were released from the scaffolds during the extraction period and suggest that the incorporation of titanium dioxide nanotubes does not adversely affect the biological safety of the polyurethane sponges.

The direct Alamar Blue assay (Figure 10) demonstrated cell adhesion and proliferation and corroborated cell viability on the scaffolds.

As shown in Figure 11, all scaffolds exhibited increased cell viability during the first 7 days, indicating sustained cell viability and proliferation. Overall, the scaffolds showed good cell viability at 7 days, regardless of the TNTs content. Two-way ANOVA revealed a significant effect of culture time (+), with markedly higher fluorescence values at 7 and 14 days compared with the initial time points. Other studies have demonstrated that some ceramics, such as titanium oxide (TiO_2_) with different nanostructures, enhance cell growth rates and bone formation [64,65,66]. However, after 7 days, cell viability on the scaffold decreased except for the scaffold with 1 wt% TNTs. The decrease in the Alamar Blue signal after 7 days could be explained by cell confluence and the formation of a cell monolayer, which reduces the average metabolic activity per cell, or by the natural plateau phase of osteoblast proliferation. It is worth noting that Alamar Blue measures metabolic activity as an indirect indicator of cell viability, but it does not directly detect mineralization or osteogenic differentiation [67,68].

In addition, scaffold composition (*) significantly influenced the cellular response. TNTs-containing sponges generally exhibited higher metabolic activity than the unmodified SPU control, particularly after prolonged culture. Among the tested formulations, the SPU 1% TNTs and SPU 10% TNTs groups showed the highest fluorescence values at day 14, suggesting enhanced cell proliferation and/or metabolic activity on these materials. However, although TNTs-containing scaffolds showed higher metabolic activity, the present data are not sufficient to demonstrate a specific effect of TNTs on cell metabolism. Additional studies are therefore required to clarify their biological contribution.

SEM micrographs of cells seeded on the surface of the scaffolds at 14 days are shown in Figure 11, while Figure 8 shows the porous structure of the scaffolds before cell seeding. As can be seen in Figure 11, after 14 days of culture, SEM micrographs revealed cells attached to the scaffold surface. The cells exhibited elongated and flattened morphologies with spreading; also, it can be observed that the cells have sheet-like or leaf-like extensions (corners) attached to the surface, which could indicate cell adhesion and viability on these materials [69,70,71]. However, this kind of morphology could also be related to the sample preparation method used for SEM analysis in this work. The preliminary results for SPU and SPU/TNTs composite scaffolds demonstrated that they are not cytotoxic and are potential materials for future in vitro and in vivo testing for bone regeneration.

## 4. Discussion

The increase in studies related to tissue engineering is due to the high demand for bone tissue reconstruction, given the importance of the musculoskeletal system in daily life. A large number of materials of diverse nature have been researched and tested to repair bone when it cannot self-repair, such as in cases of genetic problems, advanced age, the patient’s health status or diseases, or the severity of fractures [72]. This search demonstrated that the chemical nature of the material is fundamental to meeting requirements such as biocompatibility, degradability, and appropriate mechanical properties. Therefore, segmented polyurethanes have been investigated for their excellent modifiability to achieve different requirements, such as adequate mechanical strength and degradation rates [73,74,75].

In this work, a segmented poly(ester-urethane) was synthesized using a two-stage condensation method. The polyurethane obtained had a molecular weight of 82,600 g/mol; it is an amorphous solid with a glass transition temperature (Tg) of −53 °C, and its degradation temperature is above 200 °C. Additionally, it exhibits a contact angle of 70°, so it is considered to have hydrophilic character, which promotes material interaction with cells and body fluid [24].

Several methods for analyzing polyurethane degradation have been studied, with the hydrolysis of ester bonds and the enzymatic cleavage of urethane groups being the most common.

Literature [44] reports a 20% weight loss after 4 months in simulated body fluid at 37 °C for similar polyurethanes without TNTs, confirming the materials’ degradation. Given that the reported bone regeneration time exceeded 6 months [76], the scaffolds obtained in this work could be suitable for further study with a view to their intended use as bone scaffolds.

Several studies on polyureas and polyurethanes have been reported in the literature [75,77], and they reported good mechanical and biological properties. Nevertheless, exploring the field of composite materials has increased in recent decades. Composite materials are a combination of two components, where one component provides the necessary chemical and mechanical properties, and the other offers biological advantages.

In the literature [28,30,78,79,80], studies on polyurethane/hydroxyapatite, polyurethane/silica, polyurethane/titanium dioxide, and more recent studies on polyurethane/carbon nanotubes are examples of composite materials that exhibit good chemical, mechanical, and biological properties. These studies demonstrate the importance of using composite materials, compared with polymers alone, as a promising approach to new materials with improved properties for tissue engineering. Also, Cristina C. et al. [81] published a review on composites of different polymeric matrices reinforced with Titania of different morphologies, observing that titania enhances mechanical, thermal, electrical, and/or biological properties.

There are few studies in the literature on polyurethane and titanium nanotube composites intended for tissue engineering [29,82]. These works used polyurethane as films or fibers, combining it not only with titania nanotubes but also with carbon nanotubes. They demonstrated that their scaffolds were a good option for promoting bone regeneration. Nevertheless, the obtaining preparation of three-dimensional scaffold-based polyurethane and titania/titanate nanotubes using transformation methods such as lyophilization followed by salt leaching to obtain porous sponges for the intended application and the study of their material properties are not reported in the actual literature. The sponges provide a three-dimensional space that mimics natural bone architecture, allowing nutrient diffusion and vascularization. Meanwhile, the titania/titanate imparts bioactivity to the material through osseointegration, a process enhanced by nanotube morphology, which increases the surface area available for cell contact and thereby promotes tissue growth. So, in this work, the polyurethane and titania/titanate nanotube composite-based scaffolds were prepared and analyzed to determine their material properties and cytotoxicity. The formation of a mixture of titania and titanate nanotubes was confirmed by SEM, showing a tubular (multiwalled) nanotube structure. Additionally, the FT-IR and XRD analyses revealed the presence of anatase and hydrated titanate phases. Furthermore, the polyurethanes show Raman peaks due to ester and amine groups from the polymer matrix and those of the titania related to the TNTs. Also, the FT-IR spectra of composites with different wt% of TNTs show the interaction of hydrogen bonds between the polyurethane functional groups and the titania nanotubes.

The degradation temperature of the composites with different proportions of TNTs (1, 5, 10 wt%) was close to that of the pure polymer, above 500 °C; the residual weight corresponds to the amount of titanium nanotubes (TNTs) added in the composite. The polymer is an amorphous solid whose glass transition temperature (Tg) changes slightly due to the presence of TNTs in the composite, as was analyzed before in the DSC results. In the thermogram of Figure 5, it is observed that pristine polymer (SPU) does not show melting points, which could be related to the fact that it is an amorphous solid. This happens when the polymer chains are disordered. The literature on polyurethanes mentions that they could be arranged in two phases when the soft and hard segment phases are aligned [44]; neither the SPU obtained nor the composites obtained in this work present phase separation.

The SPU exhibits greater tensile strength and deformation than the composites because the TNTs are randomly distributed, preventing chain slippage into the polymer.

The tensile strength values obtained for polyurethane/TNTs are similar to those reported in the literature [79] for polyurethanes and carbon nanotubes; they also analyzed that the incorporation of the nanotubes increases the stiffness but could also reduce the tensile strength. Furthermore, some reports for trabecular bone mentioned a range between 5 and 10 MPa for tensile strength [6,7].

For the thermal behavior [83] report similar thermal stability in thermoplastic polyurethanes reinforced with carbon nanotubes, where transition temperatures were not affected by minimal concentrations of carbon nanotubes. Regarding tensile strength, concentrations below 1 wt% of TNTs improve elongation at break, while at higher concentrations, tensile strength decreases.

It should also be noted that the material is designed to degrade while serving as a bridge for Tissue Engineering, which requires not only external biological agents or the design, synthesis, and modification of the materials that make up extracellular matrices, but also the design of a 3D interconnected pore morphology in the scaffold [22,84]. According to the literature, emulating the morphology of the tissue to be repaired is fundamental for its correct reproduction, which is why numerous novel transformation techniques have been investigated, and others continue to be developed.

The process used to obtain materials that emulate soft tissues, such as cartilage, is electrospinning. However, for the regeneration of hard tissues like bone, other techniques are used, such as casting, freeze-drying, or 3D printing/bioprinting. In this work, three-dimensional polyurethane/TNTs scaffolds were obtained by combining freeze-drying and salt leaching techniques, resulting in sponge-like materials with irregular pores ranging in size from 8 to 700 µm. The pores obtained by freeze-drying are the result of the sublimation under pressure of the solvents used; the solvent system used in the present work is (polyurethane/DMF (solvent)/water (non-solvent). DMF is a good solvent for the polyurethane, indicating a negative mixing energy. However, dropwise addition of water (non-solvent) creates an instantaneous local concentration gradient. This alters the DMF-polyurethane interaction parameter and the system itself, causing the polymer to separate from the liquid phase and precipitate as spheres, a process that reduces surface energy and stabilizes the system. As the water diffuses throughout the solution, its concentration decreases, restoring the interaction between the polymer and the DMF-water mixture. That binary mixture governs thermal behavior. Consequently, adding a small quantity of water to DMF reduces the mixture’s solidification point compared with pure DMF. The water enhances strong interactions that depress the freezing point, causing the mixture to begin crystallizing as a single phase at the condensation temperature.

Porosity is generated during the phase separation process induced by freezing and mass transfer, resulting in crystal formation from the binary mixture (DMF-water). As the polyurethane is insoluble in the solvent crystals, it migrates to the crystal boundaries and concentrates there, forming a polyurethane-rich phase. Then, the system is subjected to low pressure-below the triple point of water and the freeze-drying medium-causing the solid crystals to sublime directly into the gas phase. Since the polyurethane exists in the solid phase surrounding the crystals, the spaces previously occupied by the crystals become void, transforming the polyurethane into a porous network [85]. The salt leaching method is also reported that the porous obtained are the result of the washed crystals of NaCl that could create interconnected and irregular porous by the different crystal sizes and morphologies present in the salt [86]. This type of porosity is desirable for bone regeneration because trabecular bone has a similar morphology, making it an ideal host for the cells responsible for the bone regeneration process (mainly osteoblasts).

These scaffolds are designed for use in a biological environment, so their degradation products must be compatible with that environment. This study demonstrated that osteoblast-like cells grew on the surface of the materials during the testing period, exhibiting good viability and cell adhesion. The literature [87,88,89,90] reports on the potential interaction of cells with polyurethane-based compounds and carbon nanotubes, showing increased nanotopographic roughness in materials with higher content of nanotubes, as well as greater surface area and hydrophilicity. These factors enhance protein adsorption and cell proliferation. Furthermore, the expression of different osteogenic markers such as ALP, osteocalcin, and osteopontin was improved to further promote matrix mineralization and bone tissue regeneration. On the other hand, literature [91,92,93,94,95,96] has also reported on the interaction of titania nanotubes with different cells that promote bone regeneration. These studies demonstrated that the presence of these nanostructures enables protein absorption (e.g., fibronectin and albumin among others) and their nano topographic effect enhances the cellular response, they can transmit mechanical signals that activate different osteogenic markers such as Mito-gen-Activated Protein Kinase/Extracellular Signal-Regulated Kinase (MAPK/ERK), Phosphoinositide 3-Kinase/Protein Kinase B (PI3K/Akt) and the Yes-associated Protein/Transcriptional Co-Activator with PDZ-Binding Motif (YAP/TAZ), thereby promoting mineralization and bone formation. Given that TNTs have been identified as a promising option for enhancing bioactivity, biocompatibility, and osteogenic activity, among other physicochemical properties. The scaffolds obtained in this work, which exhibit a highly porous structure and biocompatible properties, hold potential for future biological assays both in vitro and in vivo aimed at tissue regeneration.

## 5. Conclusions

In this work, polyurethane, titanate/titania nanotubes and composites with 1, 5, and 10 wt.% of TNTs were successfully obtained. The chemical interaction between components was discussed mainly as hydrogen bonds between the nanostructures and the urethane groups from the polymer; the composite materials show good thermal stability and mechanical properties. Furthermore, 3D scaffolds were obtained by combining freeze-drying followed by salt leaching, resulting in a porous structure with pore sizes ranging from <20 to 700 µm, which were tested in vitro using human osteoblast-like cells, showing non-cytotoxicity and great cell viability. The results provide the initial requirement to continue the analysis for their intended application in tissue engineering.

The limitations of the present study stem primarily from the complexity inherent in the porogenesis process using the three-component system (polyurethane-DMF-H_2_O/polymer-solvent-nonsolvent), and the subsequent salt-leaching step; these factors hinder strict control over the scaffolds’ final porosity and connectivity. Given that the formation kinetics and the resulting morphology of the porous network depend on interdependent variables (composition, concentration, and the thermodynamic properties), precise control of the scaffold architecture relies on the systematic optimization of each operating parameter. Furthermore, the presence of titania/titanate nanotubes alters phase-separation kinetics, as pore nucleation and growth, as well as final pore morphology, depend on the interdependent variables. Likewise, a detailed analysis of the physicochemical phenomena governing the interface between the nanotubes and the polymeric matrix, as well as the thermodynamic stability of the resulting structural material, was considered beyond the scope of this investigation. Finally, although preliminary cytotoxicity assays suggest promising biocompatibility, material validation requires comprehensive biological evaluation (including in vitro cell differentiation and in vivo studies) to conclusively demonstrate its suitability for applications in bone tissue regeneration

## Figures and Tables

**Table 1:** Molar ratio, molecular weight, and polydispersity of poly(ester-urethane).

PCL Mn	PCL:HDI:BD (Molar Ratio)	Mw (g/mol)	Mw/Mn (PD)
993	1:2.16:1.16	82,600	1.75

## Data Availability

The original contributions presented in this study are included in the article. Further inquiries can be directed to the corresponding authors.
